# An Approach Towards Optimization Appraisal of Thermal Conductivity of Magnetic Thermoplastic Elastomeric Nanocomposites Using Response Surface Methodology

**DOI:** 10.3390/polym12092030

**Published:** 2020-09-06

**Authors:** Moayad Husein Flaifel

**Affiliations:** 1 Department of Physics, College of Science, Imam Abdulrahman Bin Faisal University, P.O. Box 1982, Dammam 31441, Saudi Arabia; mhflaifel@iau.edu.sa; 2 Basic and Applied Scientific Research Center, College of Science, Imam Abdulrahman Bin Faisal University, P.O. Box 1982, Dammam 31441, Saudi Arabia

**Keywords:** polymer composites, nanocomposites, thermoplastic, natural rubber, magnetic nanoparticles, heat transfer, thermal conductivity, melt compounding, response surface methodology, electronic packaging

## Abstract

This study investigates the optimization of thermal conductivity of nickel zinc ferrite incorporated thermoplastic natural rubber nanocomposites using response surface methodology (RSM). The experimental runs were based on face-centered central composite design (FCCD) where three levels were designated for both temperature and magnetic filler content. The analysis of variance (ANOVA) results showed that the implemented technique is significant with an F-value of 35.7 and a *p*-value of <0.0001. Moreover, the statistical inference drawn from the quadratic model suggests a saddle response behavior the thermal conductivity took when both factors were correlated. The factors’ optimal set confined within the practical range led to a thermal conductivity of 1.05 W/m·K, a value which is believed to be associated with an optimal percolated network that served as efficacious thermal pathways in the fabricated nanocomposites. These results are believed to contribute to the potential employability of magnetic polymer nanocomposites (MPNCs) in electronic packaging applications.

## 1. Introduction

In the past decade, rising demand in the ever-changing world on electronic products with high level specifications has put researchers and engineers in the face of various challenges, most important of which is the ease of heat dissipation that becomes such an imperative issue, especially when the device possesses high power density and miniaturized size [1,2,3]. For this reason, a great effort has been exerted in looking for suitable materials that can meet the performance and reliability criteria set by the electronic packaging industry [4,5,6,7,8].

Recently, there has been incessant race in exploring magnetic polymer nanocomposites (MPNCs), a subclass of polymer composites due to its multitude range of applications including biomedical and environmental [9,10,11,12,13], microwave absorption [14,15,16,17], and electronic applications [18,19,20,21]. Above all MPNCs offer themselves as one of the promising candidates in the electronic packaging arena due to the synergistic effect that comes from the inclusion of magnetic nanoparticles with good thermal conductivity and its polymeric matrix that is chosen based on its good mechanical properties, excellent thermal and electrical resistance, good processability, light weight, and low cost [22,23,24]. In our previous research works, different magnetic polymer nanocomposite systems were prepared for investigation of their performance in microwave absorption applications [25,26]. Thus, one of the themes of this work is to highlight the potential importance of such materials in the electronic packaging arena.

From another perspective, response surface methodology is a very powerful statistical tool that aims to determine the relationships amongst factors affecting a process and the outcome of interest. This is carried out by simultaneously varying several potentially factors screened earlier and confirmed to have a significant influence on the outcome response in the process. The variation of the independent factors is done through coded values from a low level to a high level that are chosen based on a scientific prior knowledge, and which is referred to as the pre-defined practical range. Compared to one factor at a time approach (OFAT), response surface methodology reduces the number of experimental runs used to formulate the empirical quadratic model and provides an efficient shortcut path for maximizing the output and hence is considered cost-effective [27].

Several researchers have employed the response surface methodology (RSM) technique and other algorithm approaches to optimize the properties of differently filled polymer nanocomposites [28,29,30,31]. Nonetheless, the research involving these intriguing techniques for investigating the properties of magnetic polymer nanocomposites is considered very scarce. For instance, Dorraji et al. utilized central composite design (CCD)-based RSM for predicting and optimizing the microwave absorption performance of a hybrid magnetic polymer nanocomposites by examining different levels of titanium dioxide nanoparticles (TiO_2_), strontium ferrite nanoparticles (SrFe_12_O_19_), and polypyrrole conducting polymer embedded in epoxy resin. Their results indicated that their model was significant with a *p*-value of <0.0001 and a high R^2^ value of 96.5%. The factors’ optimal conditions for a sample thickness of 2 mm brought about a maximum reflection loss of −15 dB in the X-band range of 9.2–10.8 GHz [32]. In another interesting study, Davodi et al. examined the adsorption capacity of magnetite dopamine nanocomposites for mercury (Hg (II)) removal from aqueous solution. For this, they used RSM with Box–Behnken design (BBD) for the optimization of Hg(II) adsorption capacity given in mg/g based on the variation of three significant parameters, i.e., pH, Hg(II) initial concentration, and contact time. They found that their model was significant with R^2^ value of 98.65% and a maximum sorption capacity of Hg(II) of 307 mg/g obtained at the optimal set conditions of pH of 5.36, Hg(II) initial concentration of 98.65 mg/g, and a contact time of 5.17 h [33]. Another recent work performed by Hamadneh et al., a prey predator algorithm trained-artificial neural network, was harnessed to predict the thermal conduction behavior of electrospun polyacrylonitrile (PAN) fibers impregnated with different hybrid nanofillers of multiwall carbon nanotubes (MWCNTs) and NiZn ferrite nanoparticles. The predicted thermal conductivity response using this approach was found to be in a good agreement with the respective measured value, with an R^2^ of 97.8% [34].

By surveying the literature to this date, no attempt has been reported on the appraisal of the influence of different variables on the heat transport properties of magnetic thermoplastic natural rubber nanocomposites using response surface methodology. Thus, this research work is the first of its kind meant to investigate the temperature, magnetic filler content, and their interaction effects on the thermal conductivity of magnetic filled thermoplastic natural rubber nanocomposites and to employ the results for finding out the optimized thermal conductivity value within the pre-defined practical range.

## 2. Materials and Methods

### 2.1. Materials and Preparation

NiZn ferrite nanoparticles with 98.5% purity and an average particle size range of 10–30 nm were bought from Nanostructured and Amorphous Materials Inc, Houston, Texas, USA. The magnetic nanoparticles have the chemical formula of Ni_0.5_Zn_0.5_Fe_2_O_4_ and were synthesized via a facile precipitation route as reported earlier [35,36,37]. On the other hand, the prepared polymeric matrix of two phases, i.e., thermoplastic natural rubber (TPNR), is composed of high-density polyethylene (HDPE) procured by Mobile (M) Sdn. Bhd., natural rubber (NR) provided by Rubber Research Institute of Malaysia (RRIM), and liquid natural rubber (LNR). The later was prepared using photosensitized chemical degradation of natural rubber under visible light, as prescribed in previous works [38,39,40,41,42].

### 2.2. Preparation of Magnetic-TPNR Nanocomposites

The preparation of the TPNR matrix started by a blending process of high-density polyethylene (HDPE), natural rubber (NR), and liquid natural rubber (LNR), in a ratio of 70:20:10, using a laboratory compounding machine (Model Thermo-Haake Rheomix 600p, TheromFisher Scientific, Waltham, MA, USA). The liquidous form of natural rubber served as a compatiblizing agent for both polymeric phases. In another experiment, the magnetic/TPNR matrix was formulated by introducing different levels of magnetic filler content of 4, 8, and 12 wt% into the same TPNR ingredients as mentioned above. The detailed description of the preparation and molding procedures was reported in previous work [36].

### 2.3. Characterization

The prepared magnetic/TPNR nanocomposites were undergone a morphological inspection using a scanning electron microscope (SEM Model LEO 1450VP, Carl Zeiss Microscopy, Jena, Germany) with an accelerating voltage of 30 kV, so as to investigate the distribution of NiZn ferrite magnetic nanoparticles within the TPNR matrix, which is believed to have an influence on the heat transport characteristics of the nanocomposites. For clear examination, the nanocomposite samples were subjected to a tensile fracture, after which the fractured spots were sputtered with a gold coating before the observation was made.

**Figure 1:**
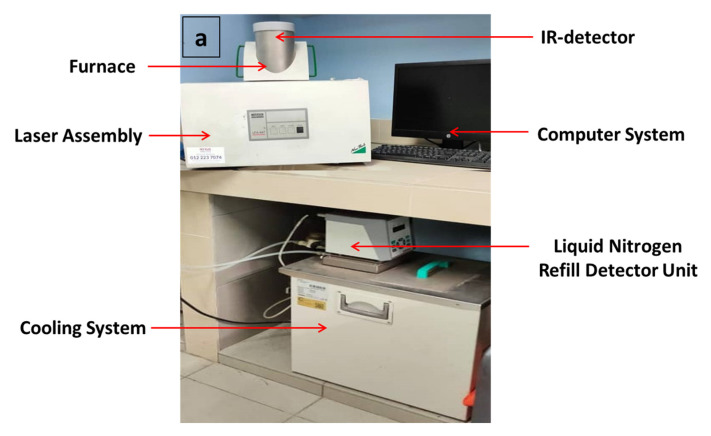

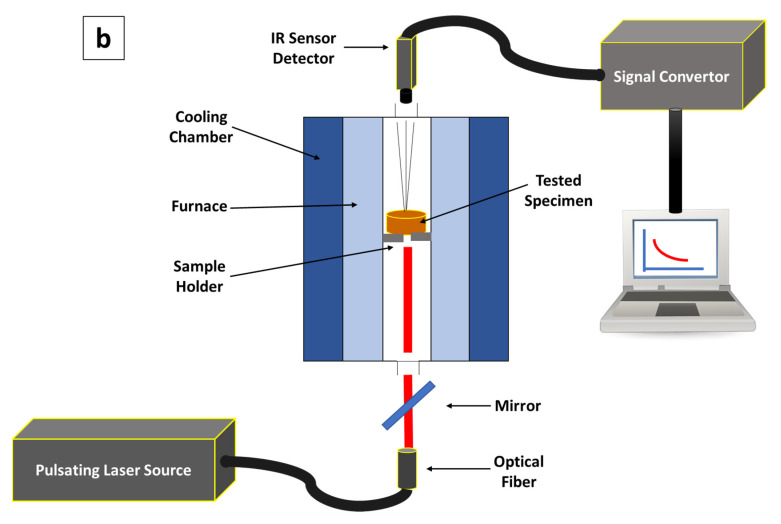
(**a**) Real experimental set-up and (**b**) schematic representation of laser flash technique used for appraising thermal conduction behavior of magnetic/thermoplastic natural rubber (TPNR) nanocomposites.

A thermal conductivity analyzer (TCA; Nanoflash NETZSCH, model LFA 44712-41) is considered a high-tech instrument that implements the laser flash method to evaluate the heat transfer properties of materials [31,43,44,45,46]. The nanocomposite samples were shaped into circular disks of 12.7 mm and 2 mm thickness and coated with graphite before commencing the test that adhered to the ASTM E1461-92 protocol as previously reported [47,48,49]. Then, the thermal conductivity measurements were performed based on face-centered central composite design. The real experimental setup and the schematic representation of the laser flash technique used for measuring thermal conduction behavior of magnetic/TPNR nanocomposites is demonstrated in Figure 1.

### 2.4. Statistical Design of Experiments

The analysis of the experimental results was based on face-centered central composite design (FCCD) using Minitab (v.18) by involving three levels of temperature and magnetic filler content. The variation of these factors was performed with coded values that range from a low level (−1) to a high level (+1) passing through the center point (0). The selection of the true uncoded values was based on previous scientific knowledge in connection to the behavior of magnetic polymer nanocomposites, which set these values as the assumed practical range. Thereafter, the experiments were designed in such a way that some were performed in the cube center point, others at the low and high levels (known as factorial points), and others were carried out at cube diagonal centers (known as star points), as given in Table 1. The experiments were conducted such as to determine the intrinsic variability and to ensure whether there is a curvature in the response surface or not.

Basically, the RSM technique uses a second-order polynomial model for obtaining the optimum response of an outcome, which is given in Equation (1) [50].

(1) $$y_{R}=\beta_{0}+\overset{k}{\underset{i=1}{\sum }}\beta_{i}x_{i}+\overset{k-1}{\underset{i=1}{\sum }}\overset{k}{\underset{j=i+1}{\sum }}\beta_{ij}x_{i}x_{j}+\overset{k}{\underset{i=1}{\sum }}\beta_{ii}x_{ii}{}^{2}$$

where *x_1_, x_2_,…x_k_* are the coded values of the input parameters, *y_R_* is the predicted response value, and *β_0_, β_i_, β_ij_*, and *β_ii_* are associated with the intercept, linear, interaction, and quadratic coefficients, respectively. On the other hand, the factors’ true values can be calculated in terms of their corresponding coded values using Equation (2) [50].

(2) $$x_{i}=\frac{T_{i}-T_{o}}{\Delta T_{io}}$$

where *x_i_* is the coded value of the factor true value, *T_i_* is the factor true value, *T_o_* is the center point of the true value, and Δ*T_io_* is the half range between the lower and higher levels of the true value.

## 3. Results and Discussion

### 3.1. Morphological Analysis

Morphological examination was carried out for the fabricated nanocomposites as it is considered a vital tool meant to determine the dispersion degree of the nanoparticles and its morphological characteristics within the polymeric host in an attempt to explain and analyze the properties, and hence be able to judge the performance of the nanocomposites. The detailed description of the elemental composition of NiZn/TPNR nanocomposites obtained from the energy dispersive X-ray spectrophotometer (EDX) is given in Table 2.

**Figure 2:**
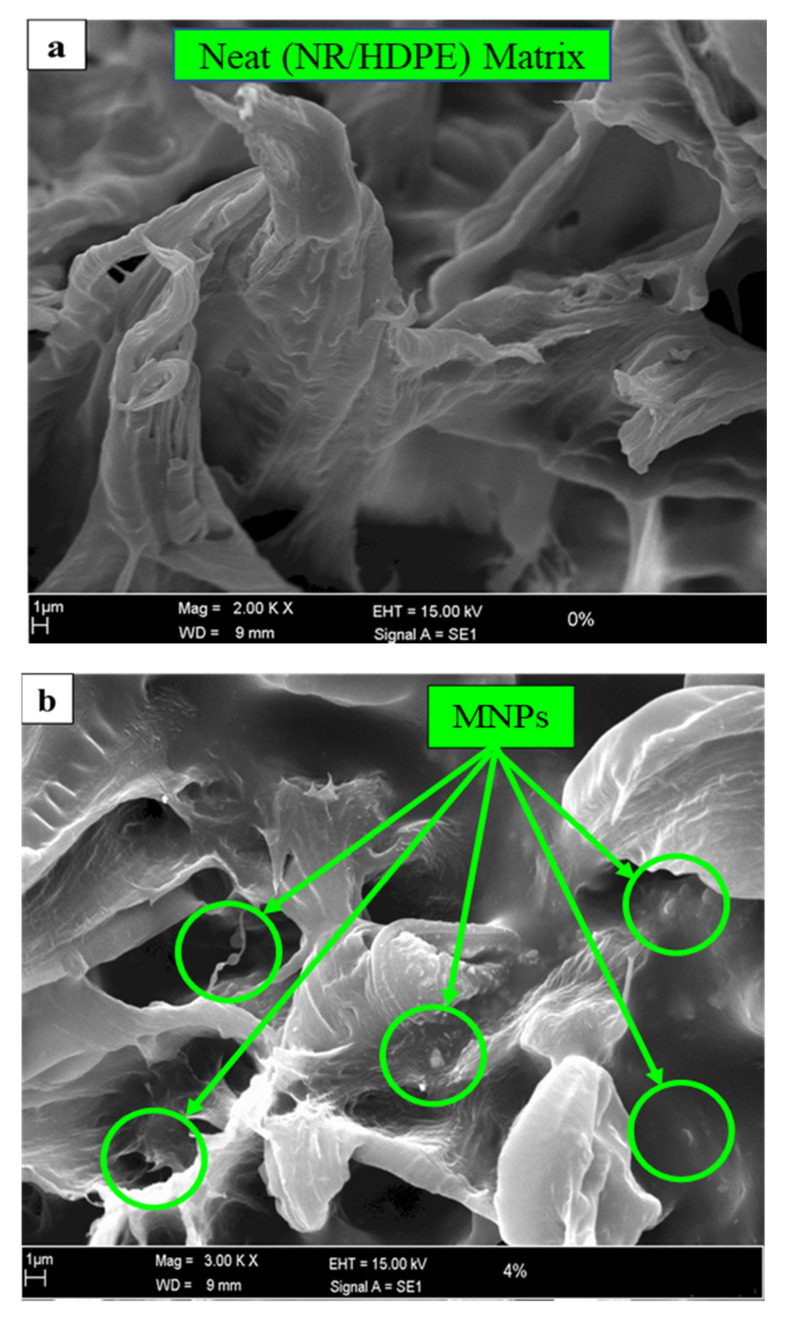

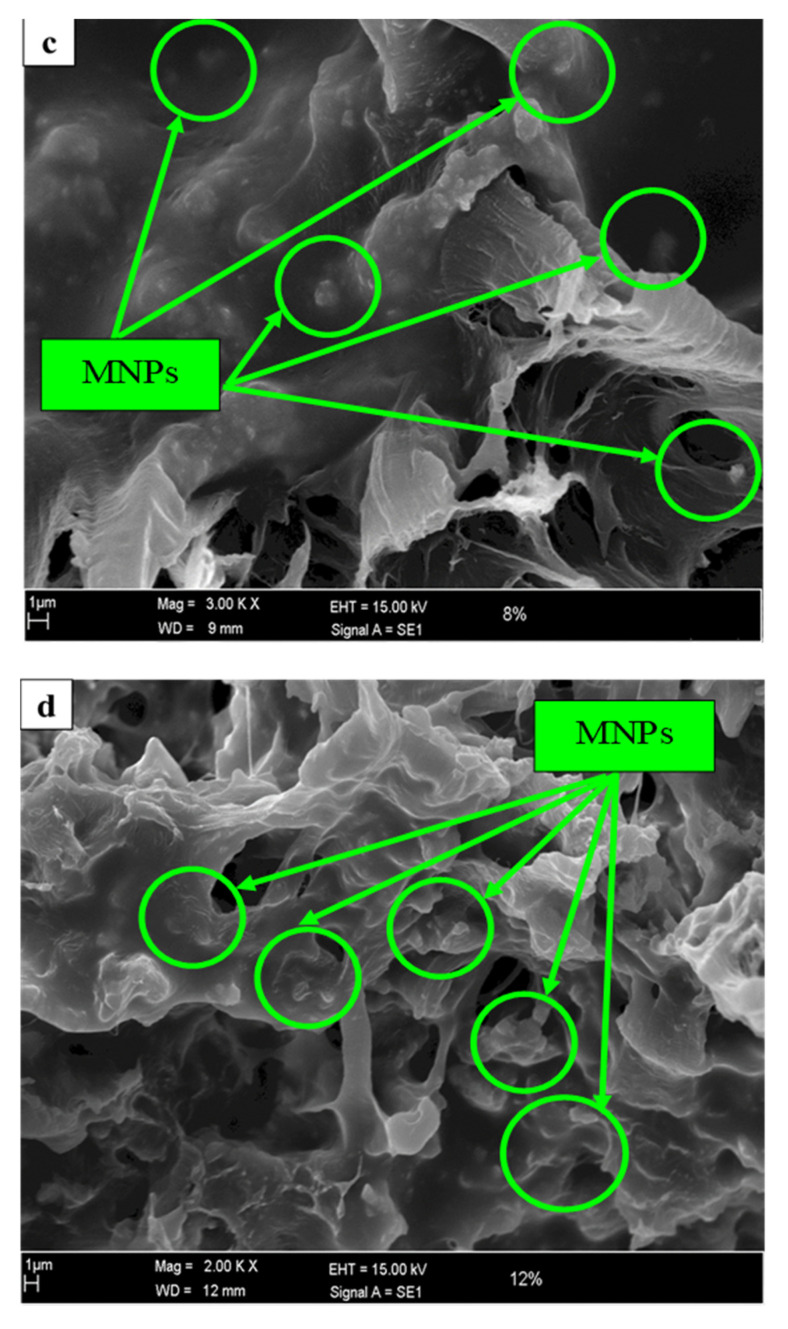
SEM micrographs of prepared nanocomposites (**a**) without magnetic filler content, and with (**b**) 4 wt%, (**c**) 8 wt%, and (**d**) 12 wt% of magnetic filler content.

Figure 2a–d demonstrates the SEM micrographs of the nanocomposite samples without and with 4, 8, and 12 wt% magnetic filler loading in the TPNR matrix, respectively. The magnetic nanoparticles in the TPNR matrix appeared to have a spherical shape with a bright-looking phase. It is well observed that for lower filler content samples, the nanoparticles distribution was far better compared to higher filled nanocomposite samples. The agglomeration effect in the 12 wt% sample was remarkably noticed, in which the nanoparticles have coalesced together forming clusters. This fact, as elaboratively discussed later in the statistical analysis section, is consistent with what several researchers have achieved in their bid to increase the amount of ceramic nanoparticles in different polymeric matrices [51,52].

### 3.2. Mathematical Model Construction and Validation

The experimental results obtained according to face-centered central composite design (FCCD) are presented in Table 3. First, for the center point of the FCCD, five replicate measurements of the thermal conductivity were carried out with a standard deviation of 0.016, which showed a minimal intrinsic variability around the center point. In addition, based on the stipulated data in Table 3, the statistical analysis was performed to correlate the temperature and magnetic filler content effects and to observe its reflection on the thermal conductivity response of the NiZn/TPNR nanocomposite samples. Accordingly, a second-order multiple regression equation in an uncoded unit was formulated, which is given as follows:

(3) $$R_{TC}=0.878-0.02514\;\beta_{T}+0.1635\;\beta_{F}+0.000158\;\beta_{T}{}^{2}-0.00824\;\beta_{F}{}^{2}-0.00011\;\beta_{T}\beta_{F}$$

where *R_TC_* is the thermal conductivity response of the NiZn/TPNR nanocomposites, *β_T_* and *β_F_* are the linear coefficients of temperature and magnetic filler content, respectively, and $\beta_{T}{}^{2}$ and $\beta_{F}{}^{2}$ are their quadratic coefficients, correspondingly.

**Figure 3:**
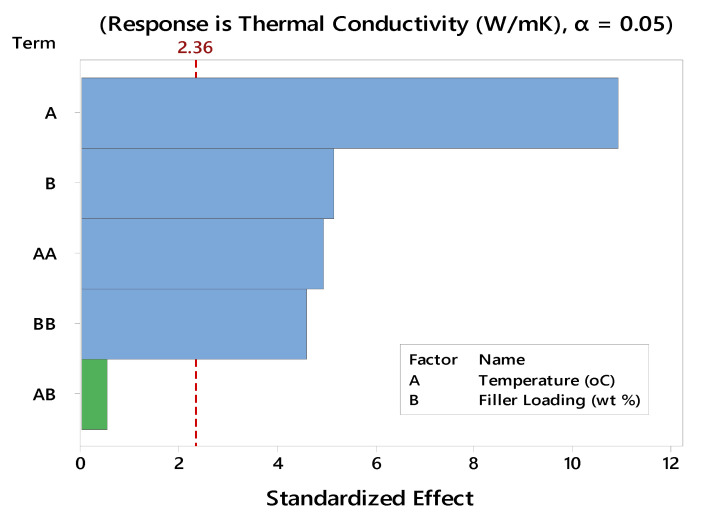
Pareto chart illustration of the standardized effect for thermal conductivity of the nanocomposites with a significant level shown by the dashed red line.

To translate the above regression formula into a more pronounced fashion, a pareto chart, as illustrated in Figure 3, was depicted, which represents the statistical significance of the influential factors and their interaction on the outcome response. It is apparently seen that the linear and quadratic terms of temperature and filler content could significantly affect the thermal conductivity, whereas their interaction effect was below the significance level and thus could be neglected when attempting to compute the predicted thermal conductivity response. Moreover, the statistical significance of the model was also evident from the ANOVA table shown in Table 4**,** with an F-value of 35.7 and a *p*-value of <0.0001.

**Figure 4:**
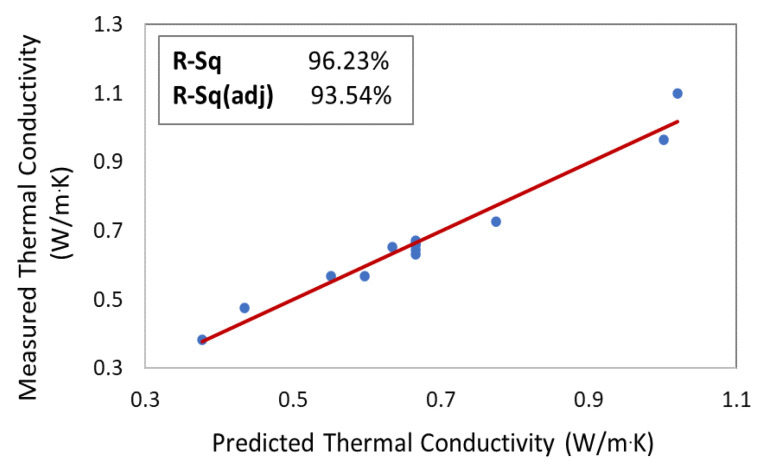
Parity plot that represents the measured thermal conductivity (blue dots) vs. the predicted one (red trendline).

For validation of Equation (1), a parity plot was depicted to comprehend how accurate the model is in predicting the response. This plot, as shown in Figure 4, demonstrates the variation of the true response as a function of the predicted one. It is evident that the measured thermal conductivity varied negligibly with the fit model, which was confirmed by the R^2^ value of 96.23%. This implies that 96.23% of the variation in the experimental thermal conductivity response could be explained by the constructed model, which is deemed a high value that made the model qualified in anticipating the response in a way that it matched very intimately with the real response value [53].

**Figure 5:**
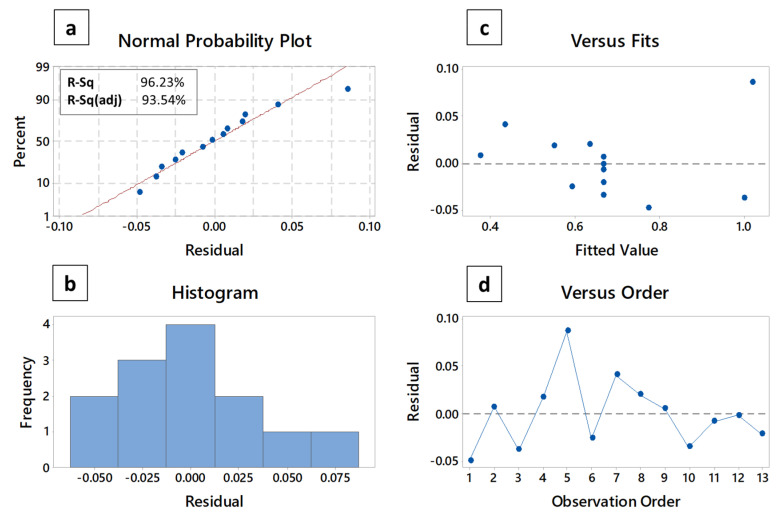
Residual plots for the thermal conductivity of the nanocomposites: (**a**) normal probability plot with residuals (blue dots) vs. ideal normal distribution (red line), (**b**) the histogram of residuals plot, (**c**) the residuals (blue dots) vs. fits (blue dashed line) plot, and (**d**) the residuals (blue dots) vs. order of data (blue dashed line) plot.

Figure 5a–d represents what is statistically well-known as residual or error plot. This four-figure illustration provides more details about the model perfection and its prediction execution aptitude. The normal probability plot, as shown in Figure 5a, hovers around the idea whether the data obtained are orderly distributed or not. It is quite clear from the depiction that most of the data formed a linear trend, which is a clear sign that the data were normally distributed. This conclusion is followed by a supportive confirmation of histogram plot, which appears in Figure 5b. The other two plots, i.e., the residual vs. fitted value and the residual vs. the observation order reveal the information of how the response and error are related. It was established that the random behavior of the data points in these kinds of plots is an indication of a good fit model [54], a fact which is typically observed in our case.

### 3.3. Response Optimization of the Thermal Conductivity of the Nanocomposites

**Figure 6:**
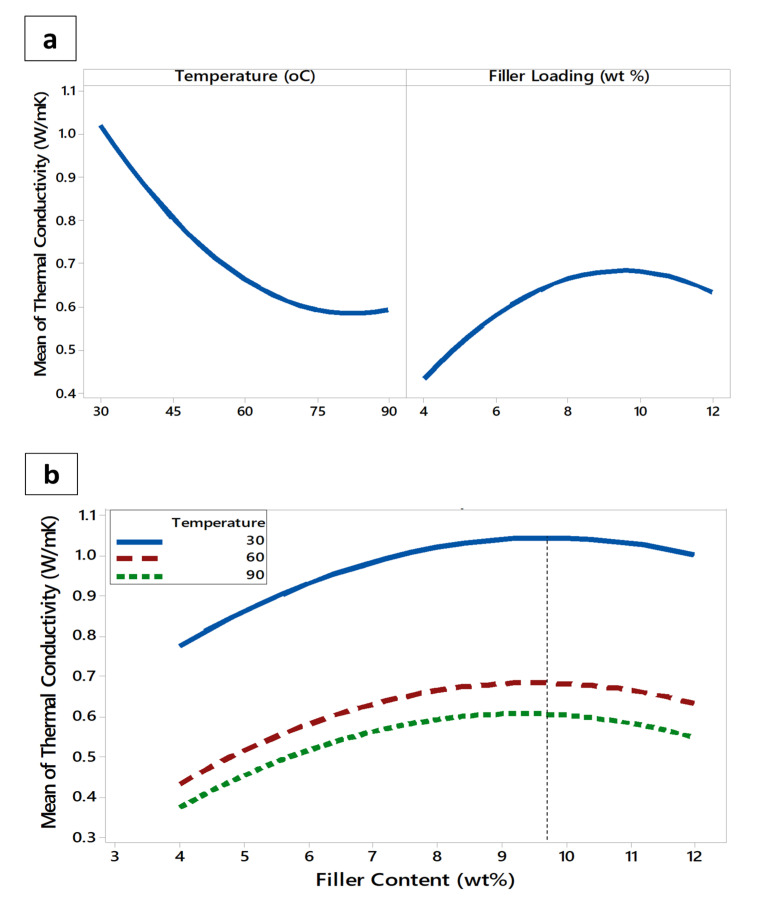
(**a**) Main and (**b**) interaction effect plots for the thermal conductivity of the nanocomposites.

Before obtaining the optimized response surface, Figure 6a,b were demonstrated to identify the significance of the main effect of the independent factors and their interaction on the outcome that can respond differently by the factors’ different levels. Consequently, if the outcome manifests a parallel response to *x*-axis, this means that there is no main effect, while its significance only shows up if the response takes a slope behavior. The higher this slope, the greater is the magnitude of the main effect. On the other hand, the interaction plot reveals how an independent factor can affect the other one and how this is reflected on the outcome response. If the factors’ effects on response are designated by parallel lines or duplicate behavior, this implies that there is no relationship, and hence no interaction exists between the factors. The interaction appears only if there is a slope variation in their curves that eventually leads to an intersection. Indeed, the higher this variation, the greater is the interaction effect [55].

Figure 6a,b show the main effect of temperature and magnetic filler content factors and their interaction effect, respectively, on the thermal conductivity response of the nanocomposites. It can be seen from Figure 6a that both factors had significant effects on the response. However, the temperature factor with higher slope has a greater main effect on the thermal conductivity as compared to the magnetic filler content. Nonetheless, this effect almost diminished when the temperature was close to 90 °C, after which a slight upturn was seen to occur due to the initiation of the phase transition in the TPNR matrix, which is certainly beyond the scope of electronic packaging application.

The other main effect plot of filler content suggests that the thermal conductivity response exhibited an increasing trend until the optimum filler content of 9.7 wt% was reached, which is believed to be associated with the optimum percolated network that served as efficient thermal pathways, as previously reported [56,57]. These results were ascertained by the morphological behavior of the magnetic nanoparticles that formed agglomerated clusters when their loading increased in the TPNR matrix, as evidenced in Figure 2d. The agglomeration effect for 12 wt% sample is believed to have increased the thermal conductive networks within the TPNR matrix, a phenomenon that had an adverse effect on the thermal conduction property, reducing thereby its value [58]. From another perspective, Figure 6b illustrates a cloning behavior the interaction curves adopted, which explicitly implies that the interaction between temperature and filler content and its reflection on the thermal conductivity response was extremely trivial.

**Figure 7:**
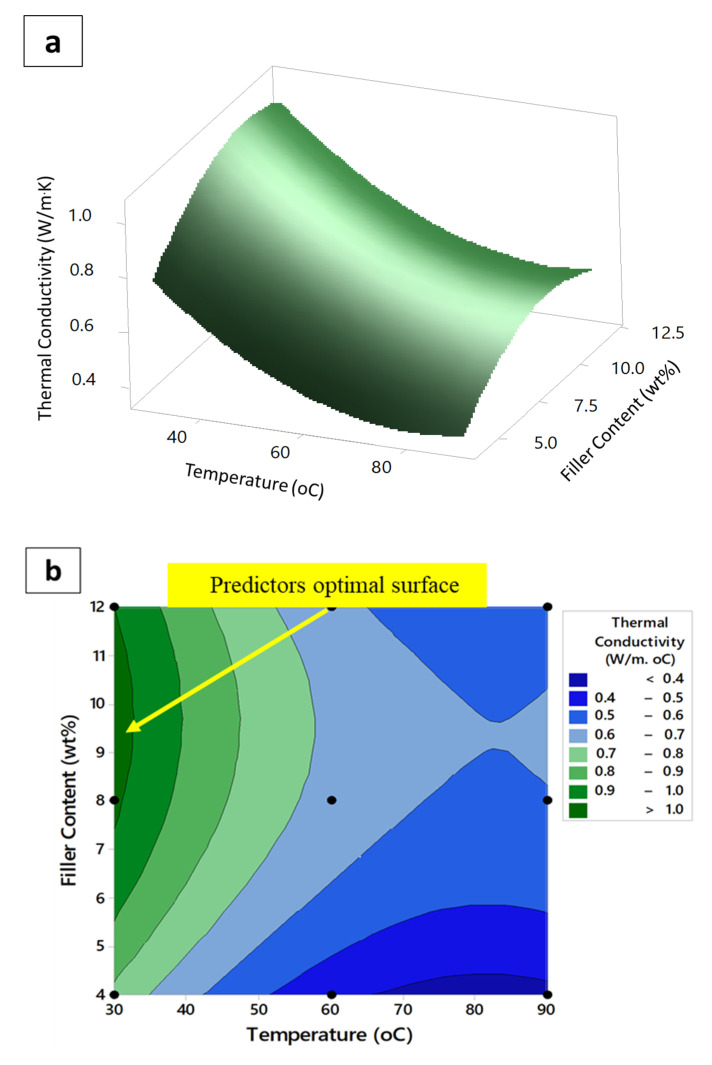
(**a**) Surface and (**b**) contour plots for the thermal conductivity of the nanocomposites vs. magnetic filler content and temperature.

Figure 7a,b demonstrate the 3D surface response and the 2D contour plots of the thermal conductivity as a function of temperature and magnetic filler content, respectively. It is apparent that the thermal conductivity took a saddle response behavior, and its value decreased with temperature for differently filled samples. This is believed to be due to the increase of structural defects in the nanocomposites with temperature, which prompted attenuation of the vibrational amplitude of the phonons via boundary scattering mechanism, and hence the observed decrease in thermal conductivity response with temperature [59,60]. As suggested by the model, the thermal conductivity responded positively only after a temperature of 90 °C was crossed, which was previously assumed to be the pre-defined practical extreme after which the segmental polymeric chain of the TPNR matrix is believed to become untangled. This fact resulted in expediting heat transfer via phonons, and hence the justified increase in thermal conductivity after this limit [23,61]. On the other hand, the optimum magnetic filler content was confirmed to be 9.7 wt%, as evidenced by both plots.

### 3.4. Experimental Verification of the Predicated Optimum Condition

The validation of the predicted optimal condition of thermal conductivity response of the nanocomposites was carried out via three experimental runs. The predicted optimal conditions of temperature and filler content of 30 °C and 9.7 wt%, respectively, were used in these experiments. The thermal conductivity average value was found to be 1.11 W/m·K, which indicates a variation of 4.8% from the predicted optimal condition, as stipulated in Table 5. This indeed implies that RSM with FCCD is a vigorous and efficient technique in optimizing heat transport characteristics of magnetic polymer nanocomposites.

## 4. Conclusions

The main theme of this work was to predict and optimize the thermal conductivity of NiZn/TPNR nanocomposites using response surface methodology with the FCCD method. First, a second-order polynomial model was established, which predicted the thermal conductivity response to a high level of accuracy. Subsequently, the optimization of thermal conductivity of the nanocomposites represented by a response surface was implemented based on the variation of values set of temperature and magnetic filler content. The ANOVA table indicated that the designed model was significant with an F-value of 35.7 and a *p*-value of <0.0001. Further, the obtained results clearly revealed a good agreement between the constructed model and the experimental data with an attained R^2^ value of 96.23%, a fact which was also evidenced from the parity and residual plots. The predictors’ optimal set of temperature and magnetic filler content was determined to be 30 °C and 9.7 wt%, respectively, which provided a maximum thermal conductivity response of 1.05 W/m·K. The predictors’ optimum conditions were experimentally validated for the thermal conductivity response, which reported a variation of less than 5% when compared to its predicted value. This indeed proves the capability of RSM with FCCD as an excellent optimization tool for this study and other prospective work.

## Figures and Tables

**Table 1:** Face-centered central composite design (FCCD) used for the optimization of temperature and filler content independent factors with coded values.

Sample ID	Point Type	Temperature	Filler Content
1	Factorial	−1	−1
2	Factorial	1	−1
3	Factorial	−1	1
4	Factorial	1	1
5	Axial	−1	0
6	Axial	1	0
7	Axial	0	−1
8	Axial	0	1
9	Centre	0	0
10	Centre	0	0
11	Centre	0	0
12	Centre	0	0
13	Centre	0	0

**Table 2:** Elemental composition of differently loaded NiZn/TPNR nanocomposites.

Element	4 wt%		8 wt%		12 wt%	
	wt%	Atomic %	wt%	Atomic %	wt%	Atomic %
C	66.57	76.85	47.39	68.93	35.61	54.72
O	24.18	20.96	19.24	21.01	29.75	34.32
Fe	5.19	1.29	21.82	6.83	20.36	6.73
Ni	1.74	0.41	4.98	1.48	6.09	1.91
Zn	2.32	0.49	6.58	1.76	8.19	2.31

**Table 3:** Randomized experimental results obtained according to face-centered central composite design (FCCD).

Sample ID	Coded Temperature	Coded Filler Content	Temperature (°C)	Filler Content (wt%)	Thermal Conductivity (W/m·K)
1	0	0	60	8	0.6706
2	1	1	90	12	0.567
3	−1	−1	30	4	0.7268
4	0	−1	60	4	0.4739
5	−1	1	30	12	0.9641
6	0	0	60	8	0.6639
7	0	0	60	8	0.6438
8	0	1	60	12	0.6535
9	0	0	60	8	0.6304
10	−1	0	30	8	1.064
11	1	−1	90	4	0.3824
12	0	0	60	8	0.6572
13	1	0	90	8	0.5683

**Table 4:** ANOVA table which represents the statistical significance of the data designed according response surface methodology (RSM) with the FCCD method.

Source	Degrees of Freedom	Adjusted Sum of Square	Adjusted Mean of Square	F-Value	*p*-Value
Model	5	0.408995	0.081799	35.74	0.000
Linear	2	0.333196	0.166598	72.79	0.000
Temperature (°C)	1	0.272896	0.272896	119.24	0.000
Filler Loading (wt%)	1	0.060300	0.060300	26.35	0.001
Square	2	0.075104	0.037552	16.41	0.002
Temperature (°C) * Temperature (°C)	1	0.055523	0.055523	24.26	0.002
Filler Loading (wt%) * Filler Loading (wt%)	1	0.048024	0.048024	20.98	0.003
2-Way Interaction	1	0.000694	0.000694	0.30	0.599
Temperature (°C) * Filler Loading (wt%)	1	0.000694	0.000694	0.30	0.599
Error	7	0.016020	0.002289	-	-
Lack-of-Fit	3	0.014979	0.004993	19.18	0.008
Pure Error	4	0.001041	0.000260	-	-
Total	12	0.425015	-	-	-

**Table 5:** Experimental validation of predicted thermal conductivity response of the nanocomposites based on the predicted optimal conditions of temperature and magnetic nanofiller.

Temperature Optimal Value (°C)	Filler Content Optimal Value (wt%)	Optimized Experimental Thermal Conductivity (W/m·K)	Optimized Predicted Thermal Conductivity (W/m·K)	Error %
30	9.7	1.10	1.05	4.8
